# Recurrence Rates After Microvascular Decompression in Patients With Primary Trigeminal Neuralgia and Its Influencing Factors: A Systematic Review and Meta-Analysis Based on 8,172 Surgery Patients

**DOI:** 10.3389/fneur.2021.738032

**Published:** 2021-09-30

**Authors:** Fangyu Chen, Yuming Niu, Fan Meng, Pan Xu, Chao Zhang, Yingying Xue, Shishi Wu, Long Wang

**Affiliations:** ^1^Center for Evidence-Based Medicine and Clinical Research, Taihe Hospital, Hubei University of Medicine, Shiyan, China; ^2^Hubei Key Laboratory of Embryonic Stem Cell Research, Taihe Hospital, Hubei University of Medicine, Shiyan, China; ^3^Department of Histology and Embryology, School of Basic Medical Sciences, Hubei University of Medicine, Shiyan, China

**Keywords:** trigeminal neuralgia, microvascular decompression, recurrence rate, risk factors, prognosis, meta-analysis

## Abstract

**Background:** Primary trigeminal neuralgia (PTN) is known to reoccur following microvascular decompression (MVD) surgery. However, the rates and contributing factors related to PTN recurrence remain controversial. The purpose of this study was to explore the postoperative recurrence rates and related influencing factors of patients with PTN after MVD. Additionally, recurrence rates after different treatments were compared to provide guidelines for clinicians.

**Methods:** We conducted systematic reviews and meta-analyses in accordance with the preferred reporting items of the PRISMA guidelines. We searched nine databases, namely, the PubMed, EMBASE, Cochrane Library, Web of Science, CINAHL, CBM, CNKI, VIP, and Wanfang databases, from establishment to July 13, 2020, selecting for studies about the long-term postoperative efficacy of MVD in the treatment of PTN. Factors associated with higher recurrence rates after MVD and long-term postoperative results of other treatments underwent formal meta-analysis, where odds ratios (ORs) with the corresponding 95% confidence intervals (CIs) were calculated. The dose-response model was used to inspect the associations between several factors and higher recurrence rates.

**Results:** Seventy-four studies (8,172 patients, 32 case series studies, and 42 non-randomized controlled trials) were analyzed in our research. Overall, 956 out of 8,172 patients relapsed, and the pooled recurrence rate was 0.096 (0.080–0.113). Influencing factors of relatively higher recurrence rates included atypical trigeminal neuralgia symptoms, lack of nerve groove, non-arterial compression, patients who were 50–60 years old, and longer disease duration. Dose–response analysis showed that the recurrence rate had a significant trend with the published year and the follow-up time. Simultaneously, the recurrence rate of MVD treatment was much lower than that of conventional drug treatment, gamma knife surgery, percutaneous balloon compression, and radiofrequency thermocoagulation. When the surgical technique was improved or combined with partial sensory rhizotomy (PSR), the postoperative recurrence rates were significantly reduced.

**Conclusions:** Even for PTN patients who have a successful operation, ~10% of them will still relapse. This research identifies several factors that can affect the recurrence rate. Compared with other operations, MVD has a relatively lower recurrence rate. Our analysis suggests that improved surgical techniques and combining PSR and MVD will yield better results.

**Systematic Review Registration:** https://www.crd.york.ac.uk/PROSPERO/, identifier: CRD42020159276.

## Introduction

Trigeminal neuralgia (TN), also called tic douloureux or Fothergill's disease, is rare, affecting 4–13 per 100,000 people (1). The proportion of women is significantly higher than that of men, and the annual incidence rate increases with age (2). TN is a common clinical cranial nerve disease with serious neuropathic pain, and simple daily activities can also stimulate its onset (3), so it greatly affects the normal life of patients. According to the International Classification of Headache Disorders (ICHD-3), TN can be divided into three categories: classical TN, secondary TN, and idiopathic TN. Primary trigeminal neuralgia (PTN) includes classic and idiopathic TN (4). There are different theories about the etiology of PTN, and neurovascular compression (NVC) is the most accepted theory, defined as the contact between the blood vessels and the trigeminal nerve, resulting in compression (5).

Microvascular decompression (MVD) is generally recognized as the most effective way to treat PTN (6). According to the newly published guidelines, MVD is the first choice for classic TN patients whose NVC shows morphological changes (4). It is a method for the treatment of etiology in which the trigeminal nerve is decompressed of conflicting blood (7). The surgery maintains the integrity of the nerve anatomy and has no influence on the normal nerve function of the trigeminal nerve.

Although there is great success in treating TN, some patients will experience varying degrees of recurrence during follow-up even if they have good results after surgery. One large-scale formal meta-analysis conducted by Holste pointed out that 76.0% of patients report being pain-free following MVD (8). Numerous original studies have reported the recurrence rate and factors. Throughout the literature, there is marked variability in the reporting of recurrence rates after MVD, ranging from 0 to 26.6% (9, 10), mostly due to differences in the sample or center variability. Furthermore, different authors have reported distinct results on the same influencing factors. There are few comprehensive studies on the recurrence rate and its related factors. Therefore, overall recurrence rates and factors related to it remain controversial.

We conducted a comprehensive and formal meta-analysis to evaluate the recurrence rate of PTN patients and to identify its significantly related factors among the most commonly reported variables, including sex, tic side, clinical presentation of TN, presence of a nerve groove, types of offending vessels, and number of responsible vessels. In addition, we further compared the long-term prognosis of MVD with that of other treatments to provide better evidence-based guidance for clinicians.

## Materials and Methods

We conducted systematic reviews and meta-analyses in accordance with the preferred reporting items of the PRISMA guidelines (11). Our research protocol has been registered at PROSPERO (Registration Number CRD42020159276).

### Literature Search

Nine databases, namely, PubMed, EMBASE, Cochrane Library, Web of Science, CINAHL, CBM, CNKI, VIP, and Wanfang databases, were searched from establishment to July 13, 2020. We also conducted manual retrieval to obtain additional relevant articles, and there was no language restriction. The main terms included “Trigeminal Neuralgia,” “Fothergill's neuralgia,” “Microvascular Decompression,” “Recurrence,” and “Risk factor.” The synonyms of Medical Subject Headings (MESH) terms, the wild card term “^*^,” and Boolean operators “AND” or “OR” were also used to search. The detailed search strategy can be found in Supplementary Table 1.

### Inclusion and Exclusion Criteria

Two independent researchers (FYC and FM) first screened the literature according to the title and abstract. In this process, inclusion criteria and exclusion criteria were strictly followed. In the case of any dispute over the results, a third researcher (LW) was required to conduct arbitration. After excluding obviously irrelevant studies, the remaining studies were read for inclusion.

We included studies if they met the following criteria: (i) patients with primary trigeminal neuralgia; (ii) the included literature had data related to patients' pain recurrence; and (iii) types of studies: case series studies or non-randomized controlled trials (NRCTs).

Studies were excluded for the following characteristics: (i) studies did not state a clear follow-up time or the follow-up time was <1 year; (ii) patient loss at follow-up exceeded 20%; (iii) unknown or inaccurate data; (iv) multiple reports or repeated literature on the same population (in the case of republished articles, we only included the one with the most sufficient data); (v) no data of control or relapse-related influencing factors that we needed; and (vi) low-quality studies; the scores of case series studies were <6 points, and the NRCT scores were <16 points.

Recurrence was defined as pain reappearing or worsening after a period of time when the pain completely disappeared or was relieved after MVD surgery. Therefore, those patients with unsuccessful surgery were outside the scope of our study. All the patients included in our study achieved favorable results after surgery.

### Study Evaluation

The quality of observational studies was independently evaluated using the National Institutes of Health (NIH) Quality Assessment Tool by two researchers (PX and FM) (12). The tool consists of nine items for case series studies. Studies with <6 points were excluded. The quality of NRCTs was assessed by using the methodological index for non-randomized studies MINORS scale (13). The scores evaluated by MINORS ranged from 0 to 24. We excluded studies with an NRCT score of <16. Potential bias was assessed and recorded for each included methodology of study.

### Data Extraction

Two researchers (FYC and PX) independently extracted relevant data from eligible studies. The extracted variables included (i) basic characteristics of studies (author, published year, country of study, hospital level, follow-up time, sample size); (ii) number of patients and number of relapses; (iii) prognostic factors related to patients (age, duration of the disease, sex, tic side, symptom of TN, type of responsible vessel, number of responsible vessels, whether there was a nerve groove); and (iv) postoperative recurrence rate of other treatments and MVD with other improvements.

### Statistical Analysis

Our study was mainly divided into two parts: to explore the influence of risk factors on the recurrence rate after MVD and to explore the distinction in the recurrence rate between MVD and different treatments. All analyses were performed using Stata software (version 15.1, Stata Corp, College Station, TX, USA).

Categorical variables were assessed by odds ratio (OR) values with their 95% confidence intervals (CIs) and *p*-values. All statistical tests were two-tailed, and a *p*-value <0.05 represented significance. The pooled recurrence rate and 95% CIs were calculated using the Freeman–Tukey double arc-sine transformation (14). The fixed-effect model or random-effect model was selected to estimate the synthesized effect size by the heterogeneity assessment. Heterogeneity across studies was tested using Cochran's Q and *I*^2^ tests. If the probability value (*p*-value) of the Q test was <0.1, or *I*^2^ was >50%, we chose the random effects model; otherwise, a fixed effects model was used (15).

Quantitative data in our study were analyzed by dose–response analysis. We used the non-linear robust error meta-regression (REMR) model to test the dose–response relationship between risk factors and the recurrence rate, which was mainly based on inverse variance-weighted least squares regression and cluster robust error variances for dealing with the synthesis of correlated dose–response data from different studies. Related methods and Stata codes that we used can be found in the paper of Xu and Doi (16). We wanted to analyze the association between factors and postoperative recurrence rates, including publication year, follow-up time, duration of disease, and patient age.

While researching the comparison of recurrence rates between different operations and MVD, we found that the treatment increased the risk of recurrence compared with MVD when OR > 1 and *p* < 0.05.

### Meta-Regression Analysis and Subgroup Analysis

When there was significant heterogeneity, meta-regression analysis and sensitivity analysis would be conducted to explore the sources of heterogeneity from several aspects including the type of study (NRCTs vs. case series), the country of study (China and other countries), hospital grade (non-grade 3A hospital, grade 3A hospital, foreign hospitals), and preoperative treatment of the patients. We also conducted an extra meta-regression analysis of the publication year.

### Publication Bias and Sensitivity Analysis

The asymmetry of the funnel chart was examined visually and further examined by Egger's and Begg's tests by using the Stata software (17, 18). Moreover, some sensitivity analysis, including the Galbraith plot (19) and the fail-safe test (20) were conducted, in case some of the weighted analyses were with obvious heterogeneity. To evaluate the impact of each study on the overall effect, a sensitivity analysis with the leave-one-out meta-analysis was carried out by omitting one study in each turn to test the robustness of the results.

## Results

### Search Results

**Figure 1:**
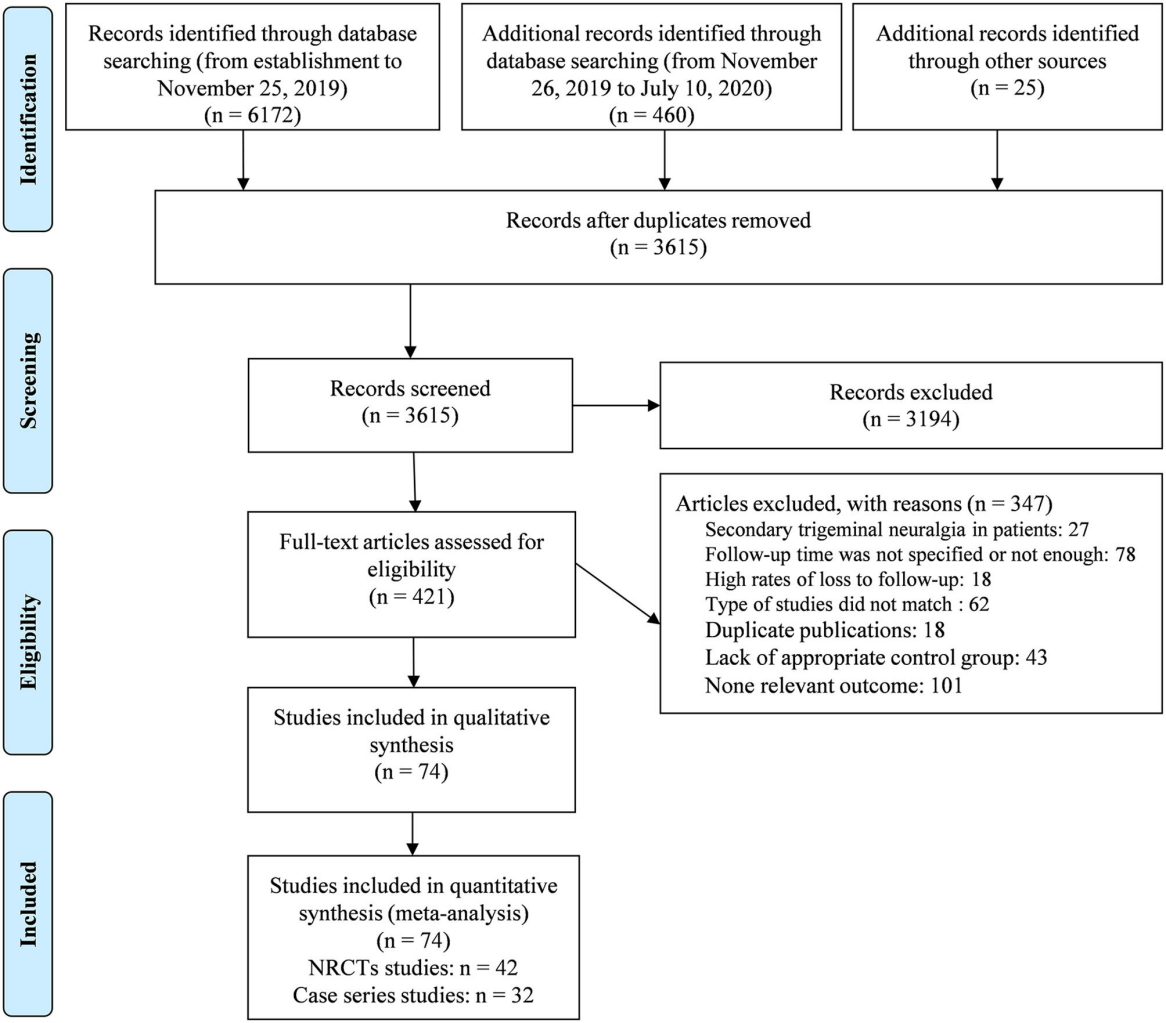
PRISMA flowchart indicating studies identified by and included in the systematic review.

The selection process is presented in the PRISMA flowchart in Figure 1. A total of 6,657 articles were retrieved (of which 25 were manually retrieved), and 3,615 articles remained after removing duplicates. After full text evaluation, 74 articles that met the predetermined search criteria were included in our meta-analysis.

### Characteristics and Quality of the Included Studies

The study characteristics are presented in Supplementary Table 2. A total of 8,172 MVD patients from 1976 to 2018 were included. It was worth pointing out that for the classification of research types, we classified all original studies related to prognostic factors as case series studies and regarded studies comparing recurrence rates between different surgical methods and MVD as NRCTs. Of the 74 studies, 32 were case series studies, and 42 were NRCTs. Regarding the six common variables, the included articles had 10 studies on sex, 6 studies on the tic side, 5 studies on clinical presentation, 4 studies on whether there was a nerve groove, 8 studies on compression vessel types, and 2 studies on single or multiple responsible vessels. All 74 studies included the recurrence rate after MVD surgery, and 42 NRCTs included the recurrence rate analysis between the MVD method and other different control treatments.

In the analysis of all 8,172 MVD patients, the proportion of female patients was much higher than that of males, with a ratio of approximately 1.336, and the incidence on the right side was much higher than that on the left side, with a ratio of approximately 1.278. The literature quality scores and potential bias in the included studies are also shown in Supplementary Table 2.

### Meta-Analysis of the Overall Recurrence Rate

As shown in Supplementary Figure 1, the recurrence rates reported among the 74 studies ranged from 0 to 26.6%, and the overall estimated recurrence rate and its 95% CI were 0.096 (0.080–0.113), which was analyzed by the Freeman–Tukey double arc-sine transformation using the random model.

The risk of publication bias regarding the recurrence rate was inspected by the funnel plot and checked with Egger's regression and a Galbraith plot. The funnel plot of the recurrence rate was symmetric (Supplementary Figure 2), and the results of Egger's test (*t* = −1.92, 95% CIs: −0.817 to 0.015, *p* = 0.059) bolstered this result. Galbraith plots (Supplementary Figure 3) found that the study of Wu et al. (10) had excessive influence on the overall estimate. We adopted sensitivity analysis (Supplementary Figure 4) to evaluate the stability of the results and found that the Wu's study truly had a significant impact on the research results, with the recalculated pooled recurrence rate of 0.094 (0.079–0.110), while leaving the study out. However, due to the large sample size that we included, the overall recurrence rate did not change too much. Moreover, the fail-safe test also confirmed the robustness of our research with the fail-safe N reaching to 1,225 using the Rosenthal approach.

### Meta-Analysis of Different Factors Related to the Recurrence Rate

Table 1 summarizes the results of the meta-analysis of the six common factors that may be related to the recurrence rate of MVD in the included studies. The heterogeneity test results showed that venous compression (*p* = 0.064) and mixed arteriovenous compression (*p* = 0.089) were significant, but the other four factors were not significant. For the weighted analysis with obvious heterogeneity, the Egger's test indicated no obvious publication bias, and sensitivity analysis confirmed the robustness of our research.

**Table 1:** Analysis of factors affecting the recurrence rates after microvascular decompression in the treatment of primary trigeminal neuralgia.

**Factor groups**	**Studies**	**N/T**	**Recurrence rates**	**Heterogeneity test**	**Model**	**OR (95% CI)**	***p***
Gender				*I*^2^ = 0.0%; *p* = 0.884	Fixed	1.050 (0.807–1.367)	0.716
Female	10	136/990	0.139 (0.111–0.169)				
Male	10	129/893	0.146 (0.112–0.183)				
Tic side				*I*^2^ = 0.0%; *p* = 0.471	Fixed	1.182 (0.841–1.661)	0.335
Left	6	66/478	0.144 (0.097–0.198)				
Right	6	100/608	0.177 (0.124–0.238)				
Clinical presentation				*I*^2^ = 0.0%; *p* = 0.899	Fixed	4.289 (1.190–15.454)	0.026
Typical	5	83/574	0.061 (0.005–0.161)				
Atypical	3	8/63	0.105 (0.031–0.205)				
Nerve groove				*I*^2^ = 0.0%; *p* = 0.831	Fixed	9.074 (4.158–19.804)	0.000
Yes	4	17/293	0.053 (0.028–0.084)				
No	4	97/394	0.248 (0.199–0.302)				
Types of offending vessel							
Artery (referent)	8	72/907	0.069 (0.035–0.113)	–	–	–	–
Vein	8	41/190	0.162 (0.038–0.332)	*I*^2^ = 47.5%; *p* = 0.064	Random	3.826 (1.779–8.227)	0.001
United	6	17/99	0.164 (0.063–0.290)	*I*^2^ = 47.7%; *p* = 0.089	Random	5.251 (1.734–15.901)	0.003
Responsible vessel				*I*^2^ = 0.0%; *p* = 0.508	Fixed	0.885 (0.257–3.046)	0.847
Single	2	11/103	0.099 (0.046–0.166)				
Multiple	2	4/50	0.077 (0.013–0.175)				

*N/T, N, number of patients who relapsed after MVD; T, number of patients who received MVD; p, p-value for differences between groups; Nerve groove, a vascular groove at the site of compression of the cranial nerve; United, mixed vascular compression*.

The pooled results showed that there were no significant differences between the groups among factors including sex, tic side, and number of responsible vessels. However, factors including the clinical presentation, nerve groove, and type of offending vessel were significantly related to the recurrence rate.

**Figure 2:**
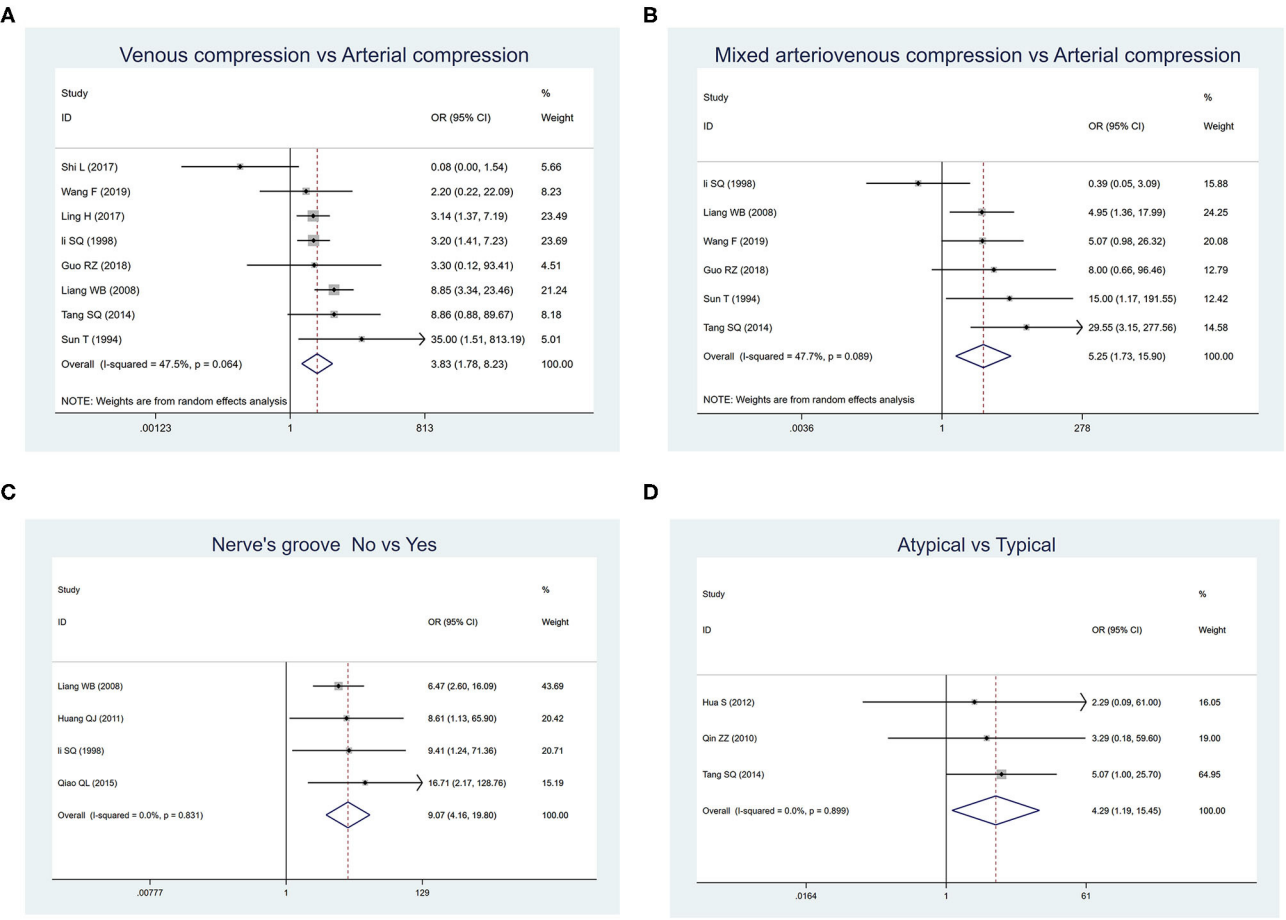
Forest plot displaying risk factors affecting the recurrence rates of primary trigeminal neuralgia after microvascular decompression. **(A)** Comparison between venous compression and arterial compression groups. **(B)** Comparison between mixed arteriovenous compression and arterial compression groups. **(C)** Comparison between groups whether there were nerve grooves or not. **(D)** Comparison between atypical and typical symptom groups.

As shown in Figure 2, the most significant factors associated with the higher recurrence rate were as follows: “venous compression” vs. “arterial compression” (OR: 3.826, 95% CIs: 1.779–8.227; *p* = 0.001), “mixed arteriovenous compression” vs. “arterial compression” (OR: 5.251, 95% CIs: 1.734–15.901; *p* = 0.003), “without nerve groove” vs. “with nerve groove” (OR: 9.074, 95% CIs: 4.158–19.804; *p* = 0.000), and “atypical TN” vs. “typical TN” (OR: 4.289, 95% CIs: 1.190–15.454; *p* = 0.026).

### Dose-Response Analysis

**Figure 3:**
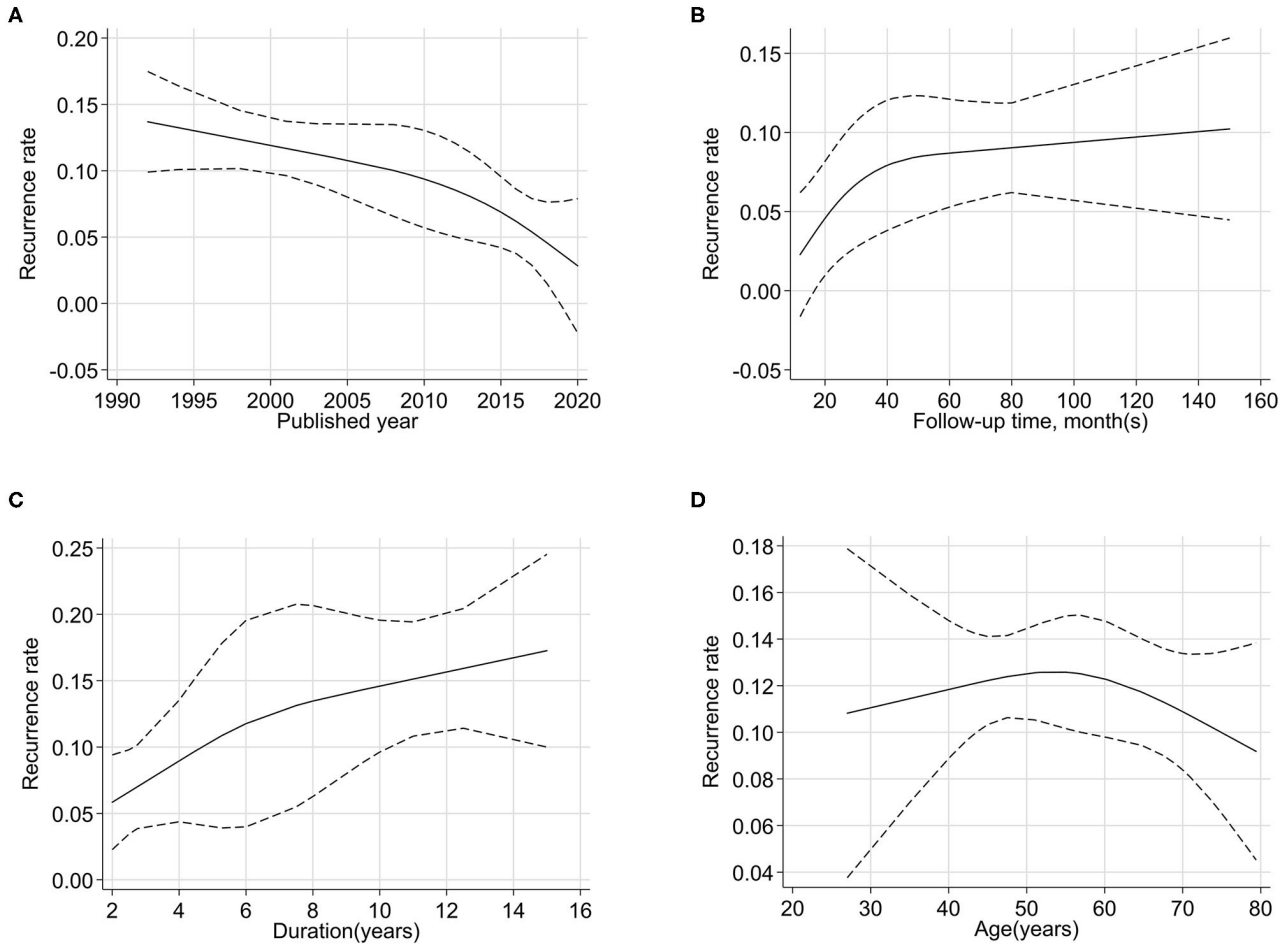
Non-linear dose–response analysis between quantitative factors [published year **(A)**, follow-up time **(B)**, duration **(C)**, and age **(D)**] and the recurrence rates of primary trigeminal neuralgia after microvascular decompression.

We explored the association of publication year, follow-up time, duration of disease, and age with the recurrence rate by dose–response analysis (Figure 3). The relationship curves suggested that more recent publications showed a lower recurrence rate. The maturity and progress of surgical technology and the development of surgical instruments contributed to a lower recurrence rate. With the increase in the median follow-up time, the recurrence rate continued to increase. The recurrence rate during the 1-year follow-up was ~2%, and it was 6% in 2 years, 8% in 3 years, and 9% in 5 years and longer.

The duration of disease and age were patient characteristics. With prolonged disease duration, the recurrence rate first increased and then stabilized. The rate of recurrence was positively correlated with disease duration. This indicated that patients would have a relatively lower recurrence rate when the surgery was performed in a timely manner. Taking 50–60 years old as a reference period, the recurrence rate of patients under 50 years old increased with age, but those over 60 years old showed a decrease in recurrence rates.

### Meta-Regression Analysis and Subgroup Analysis

Meta-regression model investigation of the “Population” and “Preoperative treatment” variable showed a significant influence on heterogeneity, while “Published year,” “Study type,” and “Hospital grade,” variables did not show a significant influence on heterogeneity.

Table 2 lists the results of the subgroup analysis. The results showed that the type of study, different countries, hospital grade, and the treatment of patients before MVD surgery have different degrees of impact on the recurrence rate of surgery. To some extent, these factors could explain the heterogeneity of this study.

**Table 2:** Subgroup analysis of recurrence rates after microvascular decompression for primary trigeminal neuralgia.

**Groups**	**Studies**	**N/T**	**Heterogeneity test**	**Model**	**Recurrence rates**	***p***
Research classification						0.090
Case series study	32	683/5,175	*I*^2^ = 85.6%; *p* = 0.000	Random	0.114 (0.090–0.139)	
NRCT	42	273/2,997	*I*^2^ = 74.5%; *p* = 0.000	Random	0.081 (0.061–0.104)	
Population						0.002
Chinese	61	646/6,315	*I*^2^ = 78.8%; *p* = 0.000	Random	0.085 (0.069–0.102)	
Others	13	310/1,857	*I*^2^= 83.1%; *P* =0.000	Random	0.154 (0.112–0.201)	
Hospital grade						0.002
Nongrade 3A hospital	9	134/965	*I*^2^ = 69.1%; *p* = 0.001	Random	0.120 (0.082–0.164)	
Grade 3A hospital	52	512/5,350	*I*^2^ = 78.6%; *p* = 0.000	Random	0.079 (0.063–0.098)	
Foreign hospitals	13	310/1,857	*I*^2^ = 83.1%; *p* = 0.000	Random	0.154 (0.112–0.201)	
Preoperative treatment						0.026
Has ever experienced MVD	2	3/99	/	Random	0.026 (0.001–0.071)	
Never experienced MVD	25	250/2,604	*I*^2^ = 80.3%; *p* = 0.000	Random	0.090 (0.064–0.118)	
Other surgery treatment	8	200/1,133	*I*^2^ = 86.0%; *p* = 0.000	Random	0.137 (0.080–0.206)	
Uncertain	39	503/4,336	*I*^2^ = 78.9%; *p* = 0.000	Random	0.096 (0.076–0.119)	

*p, p-value for differences between subgroups; NRCT, non-randomized controlled trials*.

### Recurrence Rates Vary for Different Treatments

We also conducted a meta-analysis between different types of treatment, and a significant difference in the incidence of recurrence was found between MVD and other treatments (Table 3). The heterogeneity test results showed that PSR (*p* = 0.011) and GKS (*p* = 0.089) were significant, but the other treatments were not significant. For the weighted analysis with obvious heterogeneity, the Egger's test indicated no obvious publication bias, and sensitivity analysis confirmed the robustness of our research. The pooled recurrence rate was 9.6% for MVD, 18.5% for traditional medical therapy, 12.4% for PSR, 20.9% for Gamma, 12.3% for PBC, and 11.9% for RFT. Meanwhile, the rate was 1.2% for improved MVD, 1% for MVD with PSR, and 2.3% for neuroendoscope-assisted MVD.

**Table 3:** Comparison of the recurrence rates of different treatments for primary trigeminal neuralgia.

**Treatments groups**	**Studies**	**N/T**	**Recurrence rates**	**Heterogeneity test**	**Model**	**OR (95% CI)**	***p***
MVD (referent)	74	956/8,172	0.096 (0.080–0.113)		–	–	–
Improved surgical techniques	3	2/94	0.012 (0.000–0.054)	*I*^2^ = 0.0%; *p* = 0.942	Fixed	0.205 (0.049–0.860)	0.030
Routine drug treatment	2	15/81	0.185 (0.106–0.279)	*I*^2^ = 0.0%; *p* = 0.911	Fixed	6.355 (1.760–22.945)	0.005
PSR	9	48/276	0.124 (0.042–0.232)	*I*^2^ = 59.8%; *p* = 0.011	Random	1.397 (0.585–3.337)	0.451
NSAM	2	2/84	0.023 (0.000–0.072)	*I*^2^ = 0.00%; *p* = 0.880	Fixed	0.275 (0.055–1.363)	0.114
MVD + PSR	5	4/168	0.010 (0.000–0.040)	*I*^2^ = 0.0%; *p* = 0.856	Fixed	0.188 (0.071–0.496)	0.001
GKS	8	99/611	0.209 (0.078–0.379)	*I*^2^ = 61.5%; *p* = 0.011	Random	3.238 (1.580–6.634)	0.001
PBC/PMC	6	51/382	0.123 (0.035–0.249)	*I*^2^ = 0.0%; *p* = 0.601	Fixed	1.657 (1.023–2.683)	0.040
RFT	6	59/483	0.119 (0.091–0.151)	*I*^2^ = 0.0%; *p* = 0.796	Fixed	5.385 (2.839–10.215)	0.000

*N/T, N, number of patients who relapsed after the treatment; T, number of patients who received the treatment; p, p-value for differences between other treatments with MVD; MVD, microvascular decompression; PSR, partial sensory rhizotomy; NSAM, neuroendoscope-assisted MVD; GKS, gamma knife surgery; PBC, percutaneous balloon compression; PMC, percutaneous microballoon compression; RFT, radiofrequency thermocoagulation*.

From the results, the recurrence rates of traditional medical therapy, GKS, PBC/PMC, and RFT were considered statistically higher than those of simple MVD, while MVD combined with PSR or improved surgical techniques had much better prognostic results than simple MVD.

## Discussion

In the research including 74 studies with 8,172 MVD patients, we had a sufficient sample size to analyze the influence of various factors as well as different types of treatment on their recurrence rates. We showed that the recurrence rate was approximately 10%, and characteristics including atypical TN symptoms, no nerve groove, non-arterial compression, and longer disease duration were factors that contributed to a higher recurrence rate. In addition, patients who were 50–60 years old had a relatively higher recurrence rate. To our knowledge, this research is the first comprehensive meta-analysis of the recurrence rate and its related factors after MVD. In this regard, this meta-analysis provides the most current and convincing evidence of the recurrence rate and the factors related to the impact of relapse.

The relapse rates reported were quite different (21–23). Our large-sample meta-analysis revealed that the pooled recurrence rate could be near 10%. A large series of follow-up studies by Zhong et al. (24) including 4,158 patients concluded that the rate was ~5%, but the study was excluded because of the high rate of loss to follow-up; however, we still thought this was a document worthy of reference. Meanwhile, Galbraith plots found that the study of Wu et al. (10) had excessive influence on the overall estimate; the research included TN patients with mixed presentation (atypical TN symptoms can appear along with classic TN type 1 features) who have higher recurrence rates. At present, relatively little is known about long-term prognosis of TN of mixed presentation; this study provides relevant evidence.

The research of Li pointed out that the reason for the better prognosis of MVD patients may be related to the shorter duration, older age of pain onset, limited distribution, arterial compression, and complete decompression (25). The research of Barker predicted four factors related to a higher recurrence rate after microvascular decompression: female sex, preoperative symptoms lasting more than 8 years, intraoperative venous decompression, and the lack of immediate postoperative cessation of tic after surgery (23). The predictive model for pain recurrence in the study of Theodosopoulos was age younger than 53 years at the time of surgery, symptoms lasting longer than 11.5 years, female sex, and pain on the left side in men (26). In our research, we identified five risk factors: atypical symptoms, no nerve groove, non-arterial compression, 50–60 years old, and long duration of disease. There were, thus, some differences from previous studies.

The classification of typical and atypical TN was mainly based on the symptoms and clinical presentation of the patient. Atypical TN symptom as a high-risk factor related to recurrence were consistent with the previous literature. Tyler-Kabara reported that the prognosis of patients with typical TN was better than that of patients with atypical TN (27). Nunta-Aree also found that the type of TN was a factor that could independently predict early postoperative outcome after MVD (28).

Several previous studies had proven that venous compression was an important factor leading to recurrence (23, 29), which was also confirmed in our research. Compared with arterial compression, venous compression and mixed vascular compression led to higher recurrence rates. Among the studies we included, only one study believed that the TN recurrence rate was lower in the venous compression group than in the arterial compression group (30). The authors thought that venous compression was often closely related to the trigeminal nerve, so its separation process had more operations than simple arterial compression. However, most studies held the view that the venous blood flow was slow, the petrosal vein and trigeminal nerve root were easily closely connected, and venous vessels were not easy to fully decompress (31–33). Similarly, under mixed compression, the local anatomy was more complicated, and the difficulty of separation, isolation, and other operations increase, resulting in incomplete decompression (33).

Some scholars thought that arterial compression might be more likely to lead to nerve grooves, which caused the two factors to interact with each other (34). Additionally, other scholars believe that in patients with obvious nerve grooves, although the pressure is slightly heavier than in those without grooves, compressed blood vessels and locations can often be clearly identified and easily peeled off (35). Therefore, compared with other patients with no obvious signs, patients with obvious nerve grooves during surgery will have a lower postoperative recurrence rate.

Although most of the patients with TN were female, and the affected side was mostly on the right (36), sex and location had no influence on recurrence in our study. Five studies showed that men had lower recurrence rates than women (29, 35–39). The other studies had exactly the opposite outcome (34, 40–42). In our analysis, sex was not a factor affecting the long-term prognosis of patients. The right side was more commonly affected than the left side, but it was not a significant factor affecting the recurrence rate. For the study on the number of responsible vessels, only two studies met the inclusion criteria, so the results of the meta-analysis might not be stable enough.

In our research, the more recent publications reported relatively lower recurrence rates, which may be due to the more modern technology of MVD. By observing the dose–response relationship between the recurrence rate and follow-up time, the recurrence rate increased rapidly in the first 2 years after surgery and basically had no increase in the fifth year or longer, which was similar to other series. Sun et al. (29) reported that the majority of recurrent TNs occurred within 2 years. Life-table analysis in the research of Pollack also supported our results (43). Pamir and Peker (44) also stated that after 5 years of follow-up, patients who were free from pain were at relatively low risk for recurrence. From our results, we may conclude that if the patient did not relapse within 5 years after the operation, the probability of recurrence could be very low. Postoperative tracking was recommended for patients up to at least 5 years after surgical intervention. If the prognostic research conditions were not met, the average follow-up time after surgery should be advised to be at least 2 years.

In the study of the duration of the disease, we found that the longer the illness, the worse was the long-term prognosis of the patient. Scholars speculated that during the process of nerve remyelination and repair, the probability of abnormal conduction reoccurring increased in patients who had been sick for a long time (35). Alternatively, long-term chronic compression of blood vessels could lead to partial irreversible changes in nerve roots (41).

We performed a dose–response analysis on the age and duration of disease of the patients. In this study, we found that 50–60 years old was a period for a relatively higher recurrence rate. With age, the recurrence rate first rose and then fell. This result suggested that older people could also have a good long-term prognosis and that advanced age may act as a protective factor on the probability of recurrence. Most current studies have also shown that the postoperative prognosis of elderly patients is not worse than that of young patients. Phan et al. (45) concluded that the recurrence rates were 11.9% in elderly patients and 15.6% in young patients. Amagasaki et al. (46) thought that surgical exposure of the cerebellopontine angle was generally considered easy due to atrophy, and surgery was usually performed quickly and smoothly in elderly patients. We included four literature that had patients younger than 18 years old, but the results of subgroup analysis showed that the inclusion of <18-year old patients had little effect on the recurrence rate.

Experienced surgeons can modify the operation based on clinical findings, such as using the neuroendoscope to assist and change the size of the incision or approach. Most of the relevant clinical studies have demonstrated that these improvements could effectively reduce the postoperative recurrence rate (47–49). In the literature, we classify this modified procedure as a new surgical treatment, along with studies such as RFT and PBC, as a control for the treatment of TN by MVD alone. At present, most studies and guidelines point out that MVD is the first choice for the treatment of TN (50). Our research results also confirmed that the long-term postoperative results of MVD were significantly better than those of other treatments.

The results of the subgroup analysis indicated that different types of research were not the source of heterogeneity. Meanwhile, the recurrence rate varies with the level of the hospital. We classified hospitals into three groups: grade 3A hospitals, non-grade 3A hospitals, and foreign hospitals. Patients treated in grade 3A hospitals had a lower recurrence rate than those treated in other hospitals. A nationwide study also proved that the type of hospital was a potential confounding and prognostic factor (51). It may provide good evidence for patients to choose higher-level hospitals. This research was designed to provide clinical evidence, so we did not set exclusion criteria for the patient population, such as whether they had received other non-drug treatments before surgery. As a result, we included a wider range of the population. Some of them had received drugs before surgery, some had undergone other surgery, and some had received MVD treatment. This leads to a certain degree of difference in recurrence rates.

There were several limitations of our research to consider. First, the heterogeneity of the studies was a limitation inherent to meta-analysis. This was inevitable in mass studies, and we tried our best to find the source of heterogeneity through subgroup analysis. Second, since the data of many original studies were not publicly available, we could not determine internal factors within the literature on recurrence. Research factors might interfere with each other. For example, we do not know what the sex ratio is in the study researching the impact of age. Therefore, we cannot tell whether age causes different recurrence rates or sex itself does. In addition, which surgical procedure the patient chooses depends on the judgment of the clinician of the clinical symptoms of the patient, which would introduce some bias; all NRCTs studies were subjected to potential selection bias related to control selection. Although we compared different treatments with MVD, we could not clearly point out the difference in recurrence rates among them. We hope that there will be related research in the future.

## Conclusions

Our comprehensive meta-analysis indicated that nearly 10% of the patients who had undergone successful MVD would still relapse. Several factors, including atypical TN symptoms, no nerve groove, non-arterial compression, and longer disease duration, could result in a higher recurrence rate. The recurrence rate of patients aged 50–60 years old could be relatively higher. Compared with conventional drug treatment, gamma knife, PBC, and RFT, MVD had correspondingly lower recurrence rates. Furthermore, improving surgical techniques or combining MVD with PSR would enable a better prognosis for PTN patients.

## Data Availability Statement

The original contributions presented in the study are included in the article/Supplementary Material, further inquiries can be directed to the corresponding author/s.

## Author Contributions

FC, LW, and SW collaborated in the conception and design of the project, drafted the first draft, and modified the manuscript. LW and YN searched the databases to get relevant studies. FC and PX extracted data from eligible studies. FM, PX, and YX tested the data collection forms and evaluated the qualities of studies that were involved. LW, FC, and CZ conducted the analysis and interpreted the data. The final manuscript was read, modified, and approved by all authors. All authors had full access to all of the data in the study and took responsibility for the integrity of the data and the accuracy of the data analysis.

## Funding

This work was supported by the Cultivating Project for Young Scholar at Hubei University of Medicine (2016QDJZR23) and the Funding Project of Taihe Hospital (2019JJXM039).

## Conflict of Interest

The authors declare that the research was conducted in the absence of any commercial or financial relationships that could be construed as a potential conflict of interest.

## Publisher's Note

All claims expressed in this article are solely those of the authors and do not necessarily represent those of their affiliated organizations, or those of the publisher, the editors and the reviewers. Any product that may be evaluated in this article, or claim that may be made by its manufacturer, is not guaranteed or endorsed by the publisher.
