# Supplementary material for: Recurrence Rates After Microvascular Decompression in Patients With Primary Trigeminal Neuralgia and Its Influencing Factors: A Systematic Review and Meta-Analysis Based on 8,172 Surgery Patients
Source: Front Neurol. 2021 Sep 30;12:738032. doi: 10.3389/fneur.2021.738032 (PMC8514877; doi:10.3389/fneur.2021.738032)
Supplement: Supplementary file 1 [file Data_Sheet_1.pdf]

Supplementary Material

Supplementary Table 1. Search strategies used in the databases of PubMed and EMBASE.

Online databases	Search strategy
PubMed	((((“Trigeminal Neuralgia”[MeSH] OR “epileptiform neuralgia” OR “face ague” OR forthegill OR “Fothergill's neuralgia” OR prosopalgia OR “tic douloureux” OR “tortua facies” OR “trismus dolorificus”)) AND (“Microvascular Decompression Surgery”[MeSH] OR MVD OR “microvascular decompression”)) AND (Recurrence[MeSH] OR “Treatment Outcome” [MeSH] OR prognosis OR “curative effect*” OR “clinical effect*” OR “therapeutic value*” OR “pain rate” OR “therapeutic effect” OR Factor* OR postoperat* OR rate* OR Efficacy OR effect* OR impact OR Evaluation OR Outcome* OR value*)) NOT ((animals not (humans and animals)))
EMBASE	('trigeminal neuralgia' OR 'epileptiform neuralgia' OR 'face ague' OR forthegill OR 'fothergill* neuralgia' OR prosopalgia OR 'tic douloureux' OR 'tortua facies' OR 'trismus dolorificus') AND ('microvascular decompression surgery' OR mvd OR 'microvascular decompression') AND (recurrence OR 'treatment outcome' OR prognosis OR 'curative effect*' OR 'clinical effect*' OR 'therapeutic value*' OR 'pain rate' OR 'therapeutic effect' OR factor* OR postoperat* OR rate* OR efficacy OR effect* OR impact OR evaluation OR outcome* OR value*) NOT (rat* OR mice)

Supplementary Table 2. Characteristics of the included studies.

Author	Year	Country	Follow-up time(months)	Age (years)	Duration (years)	Male/Female	Left/Right	Preoperative treatments	N	T	Recurrence rate	Study type	Tool	Grades	Potential Bias
Sun T	1994	Japan	80(13-126)	19-84	0.25-22	20/41	28/33	Group 2	10	56	17.86%	Case series	NIH	9	Interviewer, recall, responder
Li SQ	1998	China	40.8(3-144)	30-78	0.25-30	105/82	75/112	Group 4	33	187	17.65%	Case series	NIH	7	Interviewer, recall, responder
Kondo A	2001	Japan	150(60-240)	/	/	104/177	/	Group 4	23	281	8.19%	Case series	NIH	6	Interviewer, recall, responder
Javadpour M	2003	America	61(33-110)	17-90	1-30	/	/	Group 3	9	78	11.54%	Case series	NIH	7	Interviewer, recall, responder
Li ST	2004	China	13(10-27)	35-84	0.08-15	19/43	16/46	Group 2	2	45	4.44%	Case series	NIH	7	Interviewer, recall, selection, responder
Liang WB	2008	China	24	17-79	1-12	98/121	/	Group 4	25	219	11.42%	Case series	NIH	7	Interviewer, recall, responder
Sekula RF	2008	America	48(44-52)	17-88	/	/	/	Group 4	12	48	25.00%	Case series	NIH	6	Interviewer, recall, responder
Miller JP	2009	America	24	/	/	38/106	/	Group 4	25	144	17.36%	Case series	NIH	6	Interviewer, recall, responder
Qin ZZ	2010	China	25(3-46)	31-65	0.25-7	12/20	10/22	Group 1	2	32	6.25%	Case series	NIH	7	Interviewer, recall, responder
Huang QJ	2011	China	33(6-60)	35-71	0.5-20	90/71	64/97	Group 4	31	161	19.25%	Case series	NIH	7	Interviewer, recall, responder
Hua S	2012	China	21.5(12-34)	31 -85	0.5-30	18/49	25/42	Group1	1	67	1.49%	Case series	NIH	7	Interviewer, recall, responder
Xie HW	2012	China	28.2(25.2-31.2)	40-82	0.25-27	51/92	/	Group 3	7	125	5.60%	Case series	NIH	7	Interviewer, recall, responder
Jiang WM	2014	China	24(12-36)	/	0.17-13	22/46	20/48	Group4	12	68	17.65%	Case series	NIH	6	Interviewer, recall, selection, responder
Wang TJ	2014	China	28(12-67)	26-78	0.2-30	91/110	82/119	Group 2	11	187	5.88%	Case series	NIH	6	Interviewer, recall, selection, responder
Tang SQ	2014	China	12	34-75	/	54/53	51/56	Group 2	9	107	8.41%	Case series	NIH	9	Interviewer, recall, responder
Zhou WD	2015	China	36(12-60)	33-76	0.5-18	72/112	81/103	Group 4	34	184	18.48%	Case series	NIH	6	Interviewer, recall, selection, responder
Qiao QL	2015	China	26.5(5-48)	32-65	0.58-12	58/62	60/60	Group 4	25	120	20.83%	Case series	NIH	7	Interviewer, recall, responder
Martinez-Anda JJ	2015	Mexico	36(3-120)	/	/	81/190	91/180	Group 4	44	271	16.24%	Case series	NIH	7	Interviewer, recall, responder
Zhao H	2016	China	36	32–86	0.25-26	157/253	252/158	Group 4	48	410	11.71%	Case series	NIH	7	Interviewer, recall, responder
Ling H	2017	China	27.6(12-60)	27-69	0.5-11	141/159	124/176	Group 3	37	300	12.33%	Case series	NIH	8	Interviewer, recall, responder
Shi L	2017	China	24	63.7±6.60	3-16	40/32	/	Group 4	6	60	10.00%	Case series	NIH	7	Interviewer, recall, responder
Guo XF	2018	China	22(3-48)	30-84	0.25-25	40/40	39/41	Group 2	0	80	0.00%	Case series	NIH	7	Interviewer, recall, selection, responder
Zhang ZG	2018	China	12	19-73	0.50-13	35/45	36/44	Group 4	4	80	5.00%	Case series	NIH	7	Interviewer, recall, responder
Song G	2018	China	29.43(1-77)	25-81	0.08-25	120/166	103/183	Group 4	28	260	10.77%	Case series	NIH	9	Interviewer, recall, responder
Chai KJ	2018	China	24(12-36)	36-86	0.17-30	31/42	29/44	Group 3	11	73	15.07%	Case series	NIH	6	Interviewer, recall, responder
Guo RZ	2018	China	17.28(9.6-24.96)	38-79	1-9	22/40	/	Group 2	3	62	4.84%	Case series	NIH	7	Interviewer, recall, responder
Zheng JH	2018	China	24	/	/	/	/	Group 2	36	400	9.00%	Case series	NIH	9	Interviewer, recall, responder
Wu A	2018	America	20.6(12-24)	53.3±13.3	4.87±5.77	158/301	/	Group 3	122	459	26.58%	Case series	NIH	6	Interviewer, recall, responder
Jafree DJ	2018	America	60(14.4-105.6)	46.3-70.8	2.0-8.0	71/98	53/110	Group 2	28	169	16.57%	Case series	NIH	9	Interviewer, recall, responder

Zhang WD	2019	China	31(12-48)	35-70	0.5-14	72/92	62/102	Group 2	31	164	18.90%	Case series	NIH	9	Interviewer, recall, responder
Wang F	2019	China	60	24-78	/	118/94	96/114	Group 4	7	212	3.30%	Case series	NIH	7	Interviewer, recall, responder
Mastronardi L	2019	Italy	26(20.5-31.5)	40-84	/	19/47	/	Group 2	7	66	10.61%	Case series	NIH	6	Interviewer, recall, responder
Cho DY	1992	China	75.6(6-108)	36-80	/	/	/	Group 4	53	376	14.10%	NRCT	Minors	20	Interviewer, recall, selection, responder
Walchenbach R	1994	Netherlands	77.3(8-146)	/	/	40/19	25/32	Group 3	8	50	16.00%	NRCT	Minors	19	Interviewer, recall, selection, responder
Chen JF	2003	China	24	24-87	0.25-22	102/139	/	Group 2	20	114	17.54%	NRCT	Minors	20	Interviewer, recall, selection, responder
Zhang l	2004	China	51(36-73)	70-79	/	32/22	/	Group 4	4	37	10.81%	NRCT	Minors	18	Interviewer, recall, selection, responder
Yan S	2008	China	42(12-72)	32-79	0.25-25	84/97	67/114	Group 2	15	160	9.38%	NRCT	Minors	19	Interviewer, recall, selection, responder
Linskey ME	2008	America	40.8(2.04-102)	36-92	0.33-40	33/47	33/47	Group 2	7	36	19.44%	NRCT	Minors	19	Interviewer, recall, selection, responder
Xu LS	2011	China	60	35-78	0.58-9.17	89/143	101/131	Group 4	7	80	8.75%	NRCT	Minors	19	Interviewer, recall, selection, responder
Zhu LX	2012	China	40(10-70)	/	/	/	/	Group 2	1	21	4.76%	NRCT	Minors	18	Interviewer, recall, selection, responder
Yan W	2013	China	13.5(6-18)	40-73	1-12	32/43	35/40	Group 4	10	60	16.67%	NRCT	Minors	19	Interviewer, recall, selection, responder
Wang T	2013	China	36(12-60)	45-65	/	24/36	/	Group 4	1	28	3.57%	NRCT	Minors	20	Interviewer, recall, selection, responder
Wang H	2014	China	36	30-75	/	/	/	Group 2	4	30	13.33%	NRCT	Minors	19	Interviewer, recall, selection, responder
Luo WS	2014	China	19(12-30)	19-84	0.25-25	106/112	92/116	Group 4	1	94	1.06%	NRCT	Minors	19	Interviewer, recall, selection, responder
Dai YJ	2015	China	42(12-72)	31-78	0.25-20	35/41	/	Group 4	3	38	7.89%	NRCT	Minors	19	Interviewer, recall, selection, responder
Yao CC	2015	China	21(18-24)	28-94	0.25-23	85/68	83/70	Group 2	1	28	3.57%	NRCT	Minors	17	Interviewer, recall, selection, responder
Wang L	2015	China	21(6-36)	45-77	0.25-26	114/126	75/157	Group 4	3	120	2.50%	NRCT	Minors	20	Interviewer, recall, selection, responder
Nanda A	2015	America	63.6	61.3	/	23/46	/	Group 3	4	20	20.00%	NRCT	Minors	18	Interviewer, recall, selection, responder
Lian ZH	2016	China	36	22-73	0.5-7.5	25/31	25/31	Group 4	2	28	7.14%	NRCT	Minors	20	Interviewer, recall, selection, responder
Huang ZF	2016	China	36	58. 9±10.6	6.7±7.2	27/29	23/32	Group 3	2	28	7.14%	NRCT	Minors	22	Interviewer, recall, selection, responder
Quan JJ	2016	China	15(6-24)	27-80	2-12	47/63	49/61	Group 4	9	55	16.36%	NRCT	Minors	19	Interviewer, recall, selection, responder
Wang ZZ	2016	China	21(6-36)	24-70	0.08-11	51/44	50/45	Group 4	2	50	4.00%	NRCT	Minors	19	Interviewer, recall, selection, responder
Li GP	2016	China	18	55-87	/	42/82	48/54	Group 2	11	62	17.74%	NRCT	Minors	20	Interviewer, recall, selection, responder
Qin ZY	2016	China	12	37-74	0.5-4.9	54/61	51/64	Group 4	2	61	3.28%	NRCT	Minors	19	Interviewer, recall, selection, responder
Li S	2016	China	12	30-73	0.25-20	33/39	/	Group 4	11	50	22.00%	NRCT	Minors	20	Interviewer, recall, selection, responder
Dai ZF	2016	China	24	26-89	0.25-20	90/112	/	Group 4	6	87	6.90%	NRCT	Minors	19	Interviewer, recall, selection, responder
Ning TY	2017	China	12	46-76	3-9	48/52	/	Group 2	4	51	7.84%	NRCT	Minors	19	Interviewer, recall, selection, responder
Gao J	2017	China	36	29-65	0.5-15	49/68	/	Group 2	8	52	15.38%	NRCT	Minors	20	Interviewer, recall, selection, responder
Inoue T	2017	Japan	39(12-115.2)	19–97	0.1-40	82/149	/	Group 2	11	179	6.15%	NRCT	Minors	19	Interviewer, recall, selection, responder
Wang LB	2018	China	55.8(6-72)	76-90	0.50-30	60/73	67/66	Group 2	7	80	8.75%	NRCT	Minors	20	Interviewer, recall, selection, responder
Li J	2018	China	24	31-71	1-11	48/34	39/43	Group 4	8	41	19.51%	NRCT	Minors	19	Interviewer, recall, selection, responder
Li LL	2018	China	26.11(19-37)	38 -79	0.42-17	45/62	46/61	Group 2	4	71	5.63%	NRCT	Minors	20	Interviewer, recall, selection, responder
Sun HS	2019	China	12	56.25±8.34	5.47±1.31	34/48	42/40	Group 4	7	41	17.07%	NRCT	Minors	19	Interviewer, recall, selection, responder
Li BL	2019	China	12	25-78	0.8-11	41/31	/	Group 4	1	36	2.78%	NRCT	Minors	19	Interviewer, recall, selection, responder
Cui YK	2019	China	12	38-74	0.58-8	39/51	58/32	Group 4	7	45	15.56%	NRCT	Minors	19	Interviewer, recall, selection, responder
Wen X	2019	China	19(12-60)	42-87	0.5-33	89/136	92/133	Group 2	12	109	11.01%	NRCT	Minors	19	Interviewer, recall, selection, responder

Zeng SY	2019	China	12	69.3±15.6	20.1±6.2	24/30	30/24	Group 4	1	36	2.78%	NRCT	Minors	21	Interviewer, recall, selection, responder
Wang XZ	2019	China	12	30-73	0.25-20	48/43	/	Group 4	0	45	0.00%	NRCT	Minors	19	Interviewer, recall, selection, responder
Wang HX	2019	China	14(8-24)	33-80	1-13	78/122	101/99	Group 4	2	100	2.00%	NRCT	Minors	19	Interviewer, recall, selection, responder
Xing MY	2019	China	12	40-75	/	33/37	32/38	Group 4	3	35	8.57%	NRCT	Minors	20	Interviewer, recall, selection, responder
Li JC	2019	China	15(6-24)	55-74	/	20/33	/	Group 4	0	28	0.00%	NRCT	Minors	19	Interviewer, recall, selection, responder
Zhao LF	2019	China	24	13-85	0.1-30	187/254	202/239	Group 2	1	220	0.45%	NRCT	Minors	19	Interviewer, recall, selection, responder
Cai JH	2020	China	24	62.38±8.86	2-5.25	46/67	/	Group 4	3	60	5.00%	NRCT	Minors	20	Interviewer, recall, selection, responder
Wang B	2020	China	12	27-78	2-10	47/63	/	Group 2	7	55	12.73%	NRCT	Minors	20	Interviewer, recall, selection, responder

N/T, N: Number of patients who relapsed after the treatment, T: Number of patients who received the treatment; NRCT, nonrandomized controlled trials; Group 1: Has ever experienced MVD, Group 2: Never experienced MVD, Group 3: Other surgery treatment, Group 4: Uncertain. Interviewer bias was defined as "a systematic difference between how information was solicited, recorded or interpreted," which occurred when the interviewer was not blinded. Possible recall bias occurred when the outcomes of treatment affected the patient's memory for previous events. Selection bias referred to patients dropping out or failing to follow-up or if the exposure and outcome had already occurred prior to patient selection. Response bias described the bias that occurred with self-report and surveys.

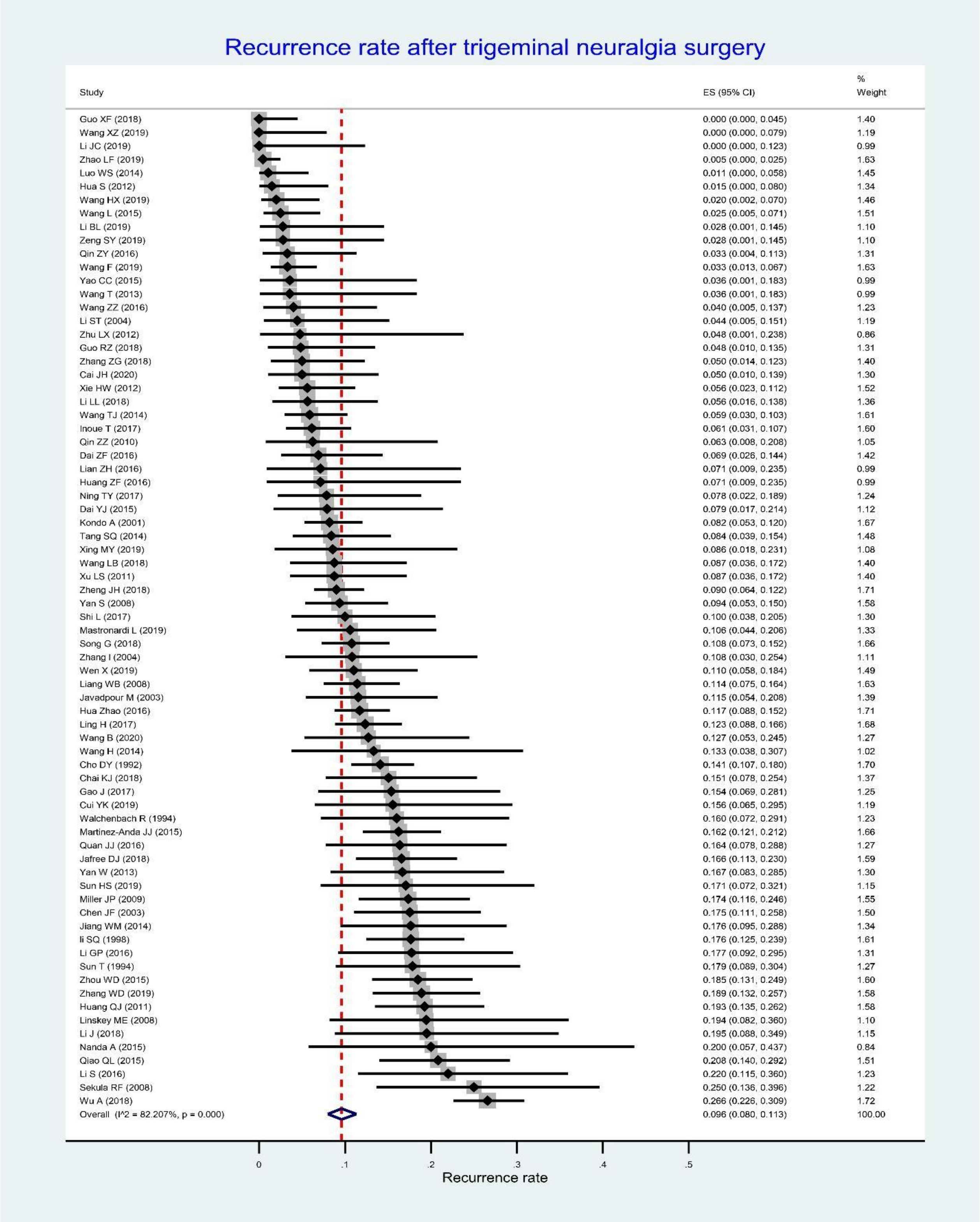

Supplementary Figure 1. Forest plot displaying the reported recurrence rates of included studies after microvascular decompression for primary trigeminal neuralgia.

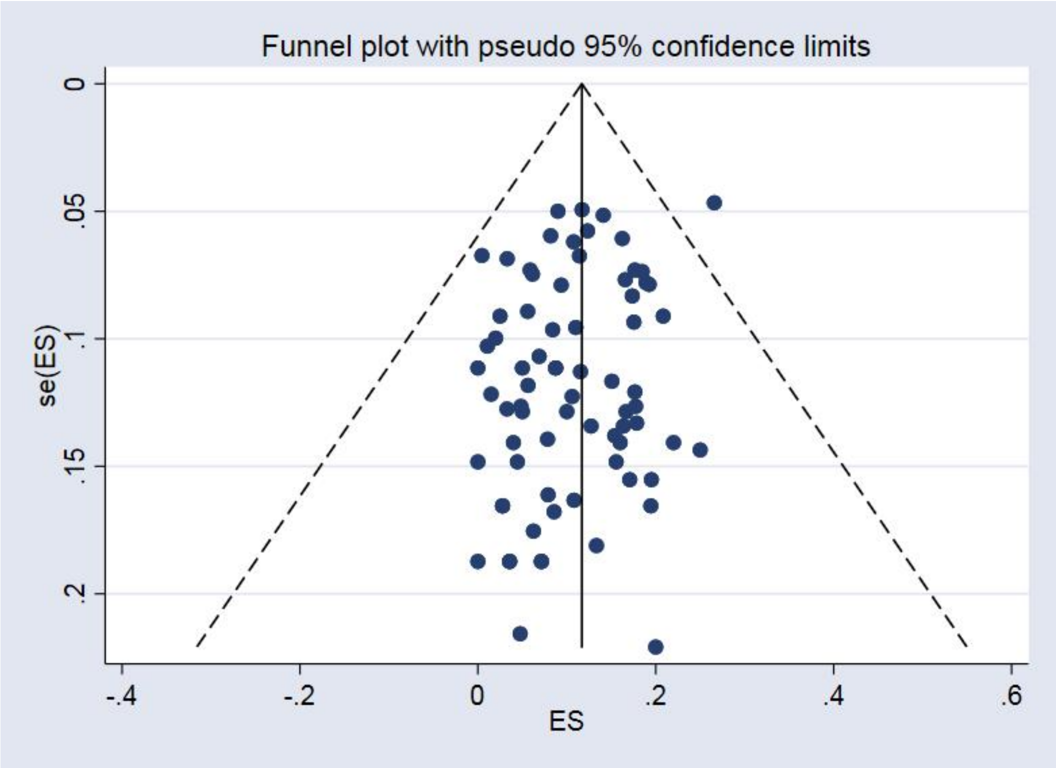

Supplementary Figure 2. Funnel plot displaying the publication bias among 74 studies included in meta-analysis.

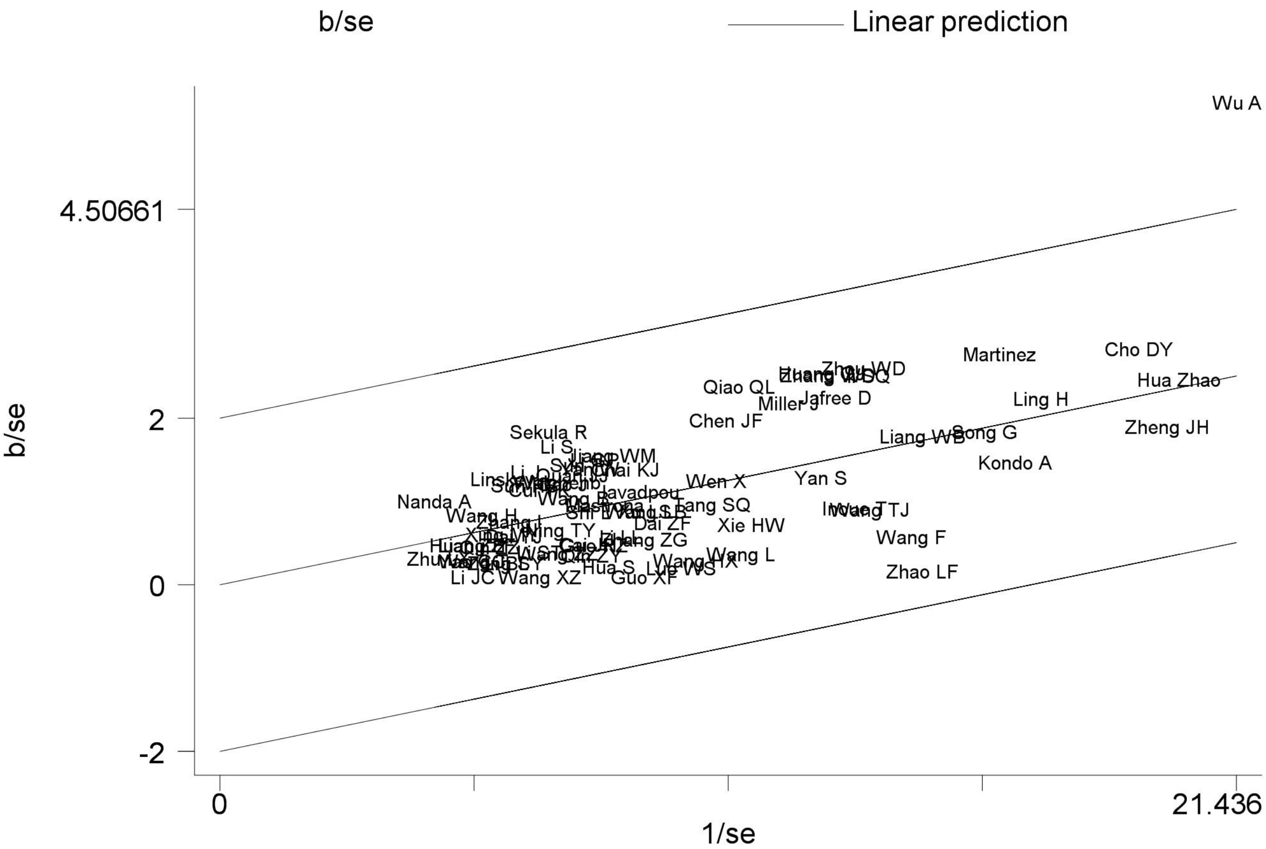

Supplementary Figure 3. Galbraith plot displaying the publication bias among 74 studies included in meta-analysis.

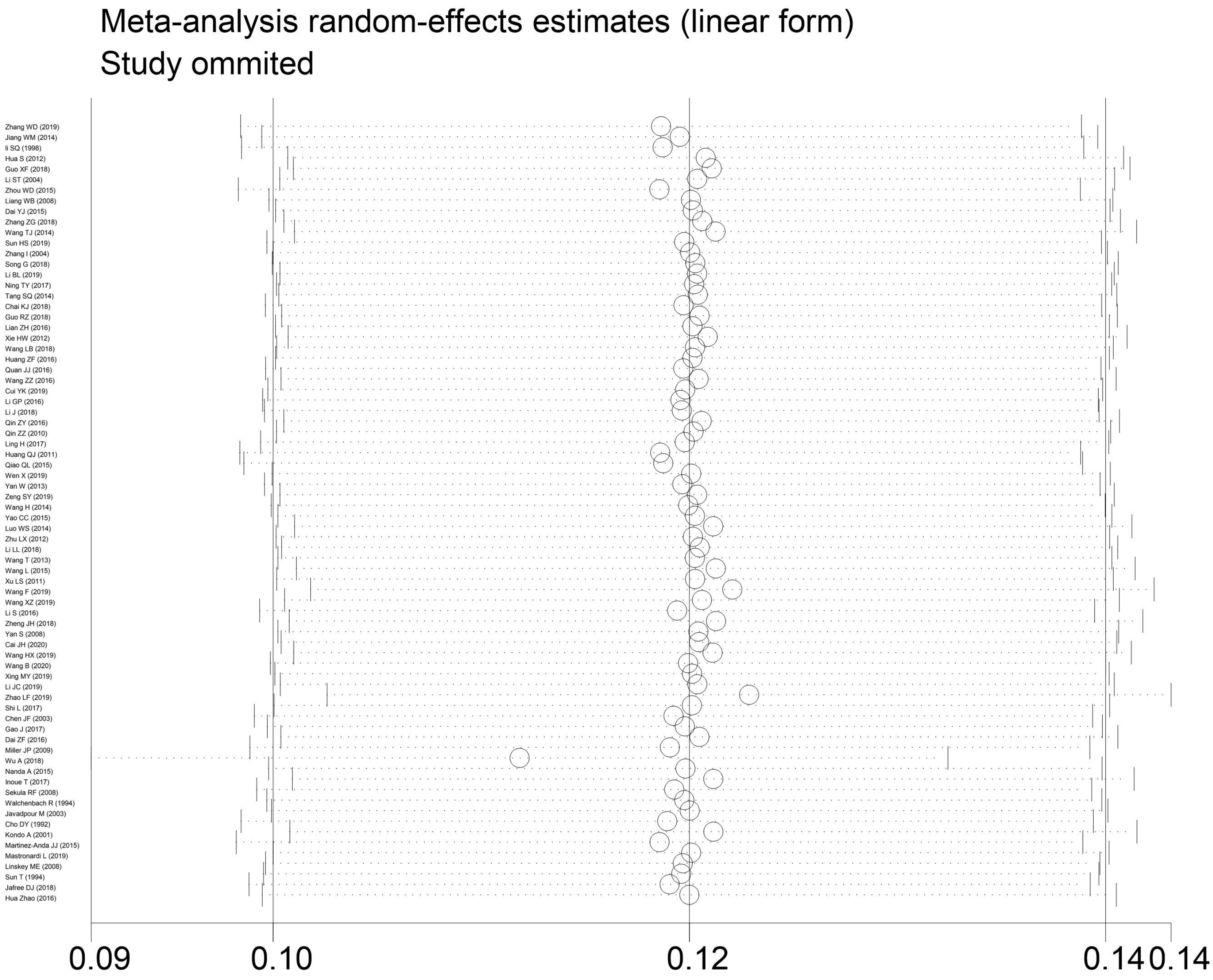

Supplementary Figure 4. Sensitivity analysis with the leave-one-out meta-analysis among 74 studies included in our research.
